# Phylodynamic Structure in the Botswana HIV Epidemic

**DOI:** 10.21203/rs.3.rs-4969814/v1

**Published:** 2024-10-18

**Authors:** Kenanao Kotokwe, Fabrícia F. Nascimento, Sikhulile Moyo, Simani Gaseitsiwe, Molly Pretorius Holme, Joseph Makhema, Max Essex, Vlad Novitsky, Erik Volz, Manon Ragonnet-Cronin, PANGEA Consortium

**Affiliations:** MRC Centre for Global Infectious Disease Analysis, School of Public Health, Imperial College London; MRC Centre for Global Infectious Disease Analysis, School of Public Health, Imperial College London; Botswana Harvard AIDS Partnership; Botswana Harvard AIDS Partnership; Department of Immunology and Infectious Diseases, Harvard T.H Chan School of Public Health; Botswana Harvard AIDS Partnership; Botswana Harvard AIDS Partnership; Brown University; MRC Centre for Global Infectious Disease Analysis, School of Public Health, Imperial College London; MRC Centre for Global Infectious Disease Analysis, School of Public Health, Imperial College London; Oxford Big Data Institute, Li Ka Shing Centre for Health Information and Discovery, Nuffield Department of Medicine, University of Oxford

**Keywords:** Phylodynamic, HIV transmission, People living with HIV (PLWH)

## Abstract

**Background:**

Studying viral sequences can provide insights into the structure of host contact networks through which the virus is transmitted. Uncovering the population structure of the HIV-1 epidemic in Botswana will help optimise public health interventions and may identify hidden sub-epidemics. We sought to determine the phylodynamic structure of the Botswana HIV-1 epidemic from viral sequence genetic data.

**Methods:**

The Botswana Combination Prevention Project (BCPP) randomly sampled 20% of households in 30 villages in Botswana between 2013–2018 and tested for HIV-1. Extensive demographic data were collected from all participants and next-generation full-genome HIV-1 sequences were generated from HIV-1 positive participants (n = 4,164), 78% of whom were on antiretroviral treatment (ART). We inferred the stage of infection (< or > 1 year) among HIV-1 cases based on nucleotide diversity and clinical data using a previously trained machine learning model. We then reconstructed time-resolved *gag* and *pol* phylogenies from sequences, other Botswana cohorts and publicly available sequences that were genetically close to those from Botswana. We statistically explored phylogenies for partitions with diverging patterns of coalescence, indicating sub-epidemics, and estimated viral effective population size through time, a measure of viral incidence, for each partition. Finally, we compared the demographic makeup, clinical and geographic characteristics across partitions using χ2, ANOVA tests and Tukey analysis.

**Results:**

We identified three partitions of time-resolved *gag* and *pol* phylogenies, revealing divergent patterns of coalescence and HIV-1 transmission. In both *gag* and *pol* phylogenies, partitions with persistent growth and transmission were characterised by lower treatment coverage and more recent infections when compared to other partitions. The Southern and South East regions of Botswana were over-represented in the fast-growing partitions.

**Conclusion:**

Our findings suggest that transmission is slowing in segments of the population that have high ART coverage. However, recent infections are over-represented in ongoing sub-epidemics. The phylodynamic structure suggests that there are districts with higher growth and prioritising these in the deployment of public health interventions might curb new infections. Nonetheless the high mobility of Botswana residents should be taken into consideration in implementing effective interventions to combat HIV-1.

## Introduction

Botswana has made great strides in combating the Human Immunodeficiency Virus (HIV) epidemic by becoming one of the first countries to reach the UNAIDS 2020 90-90-90 targets, in 2016 (1, 2). The targets called for 90% of people living with HIV (PLWH) being aware of their status, 90% of those diagnosed to be on antiretroviral treatment (ART) and 90% of those on treatment to be virally suppressed (1). The exceptional treatment programme maintained over the years (1, 2) has led to a 36% decline in HIV incidence in Botswana since 2010 (3). Nonetheless, Botswana reports one of the highest HIV prevalences in the world, 20.7% in 2019 (4). There is a need for policies that deploy public health interventions towards hard-to-reach populations where HIV cases may be concentrated.

The evolution of HIV occurs on the same timescale as its epidemiological spread (5) and thus, the former can provide insights into the latter. HIV is highly diverse within a single host and across the population due to frequent replication errors (5). The high mutation rate of HIV results in a consensus HIV genetic sequence that is almost unique to each infected individual (5). HIV genetic data can be analysed to provide insights into transmission patterns at the population level, including revealing demographic histories and estimating epidemiologic parameters that are obscured from detection by traditional epidemiological methods such as contact tracing (5, 6, 7, 8).

For example, statistical tests applied to large time-resolved viral genealogies can rapidly detect whether transmission is governed by uniform demographic and epidemiological processes (5). Here we used statistical tools to uncover the phylodynamic structure of the Botswana HIV epidemic through systematically and randomly sampled viral genetic data.

## Methods

### Study Participants

Participants aged 16–64 years from a random sample of ~ 20% of households were enrolled in a pair-matched community-randomized trial: the Botswana Combination Prevention Project (BCPP) study (1 9, 10). The BCPP study evaluated whether multiple prevention interventions (earlier ART initiation, strengthened male circumcision and enhanced HIV testing) could lead to a reduction of population-level cumulative HIV-1 incidence (9). Fifteen villages were assigned to the intervention group and 15 villages to the standard-care group between 2013 and 2018, as previously described (9). Blood specimens, demographics and clinical characteristics were collected. Data analysed here include viral sequences, age, sex, residency, inferred stage of infection, sample date and treatment status.

The BCPP study was approved by the Botswana Institutional Review Boards; Human Research Development Committee (HPDME 13/18/1) (the Institutional Review Board based at the Botswana Ministry of Health and Wellness) and the Center for Disease Control and Prevention (Protocol 6475). The study is registered at ClinicalTrials.gov Clinical trial number: NCT01965470 registered on 30/10/2013. All participants provided written informed consent for study involvement. Participants who were aged 16–18 years provided written consent with parents or guardians providing written agreement. The data and collection procedures were performed in accordance with the Declaration of Helsinki.

### Near Full-Length HIV-1 Genotyping

Viral sequences from PLWH were genotyped using a long-range HIV genotyping protocol as described (11). Most of the participants (78%) were receiving ART and were virologically suppressed, defined as having ≤ 400 copies/mL (11), and consequently, proviral DNA was predominantly used for amplification (94% of participants). Next Generation Sequencing (NGS) was carried out by the BioPolimers Facility at Harvard Medical School (https://genome.med.harvard.edu/) and through collaboration with the PANGEA HIV consortium (https://www.pangea-hiv.org/) (12, 13) using Illumina platforms MiSeq and HiSeq (13, 14). The majority consensus was assembled from NGS for each BCPP participant. For this study, we analysed 4,164 BCPP HIV sequences. Whole genome sequences were aligned, and the two main genes (*gag* and *pol*) alignments extracted using in-house script. HIV sequences and basic demographic and clinical data are available when requested from the PANGEA consortium (https://www.pangea-hiv.org/). The BCPP data and protocols are publicly available (https://data.cdc.gov/Global-Health/Botswana-Combination-Prevention-Project-BCPP-Publi/qcw5-4m9q).

### HIV-1 Subtyping

REGA HIV-1 Subtyping tool v.3 (15) was used to identify the subtype of the near full-length HIV consensus sequences. We only included HIV-1 subtype C sequences in our study excluding < 1% of sequences found to be of other subtypes.

### Inference of stage of HIV infection

Deep sequencing nucleotide frequency data were available for some of the study participants (n = 1,867), their recency (< or > 1 year) was evaluated using a previously trained xgboost gradient boosting machine learning algorithm (16). The model utilizes within-host genetic diversity, demographic (age, sex) and clinical (treatment status, viral load) to calculate a probability of recency for each individual. The algorithm was previously developed for and trained on the BCPP dataset on participants with known duration of infection, based on longitudinal follow-up (17). We selected the probability threshold which maximized accuracy of prediction of infection (recent or chronic).

### Close Global References (CGR)

We included in our analysis sequences from outside of Botswana in order to improve our phylogenetic reconstruction and to investigate international linkages. We refer to these sequences as close global references (CGR). To select CGR, we generated a global BLAST (18) database consisting of all available HIV-1 sequences where location of sampling was known and did not correspond to Botswana. This database consisted of all matching sequences in GenBank combined with the PANGEA-generated sequences not yet available in GenBank. Countries included in PANGEA are South Africa, Botswana, Zambia, Uganda, Kenya, and Tanzania. Our CGR database included 786,571 sequences, around 9,000 of which were from other PANGEA sites. For each Botswana sequence, we used the BLAST tool and custom Python scripts to select the top three closest matches within the global database as previously described (19). Our final dataset sizes were: for *gag*, 5,493 sequences inclusive of 836 CGR and 493 from previous Botswana cohorts (17); for *pol*, 4,965 sequences inclusive of 448 CGR and 353 sequences from previous Botswana cohorts (17).

### Phylogenetic analysis

Two maximum likelihood phylogenies for *gag* and *pol* sequences were reconstructed using RAxML v.8.10 (20, 21) under a GTR + Γ model (22, 23).

The treedater R package version 1.0 was used with a strict molecular clock to date the phylogenies (24). The sampling dates for the Botswana cohorts were known to the day and different alignment lengths of the two genes were factored in. Year of sample was available for most CGR, and for the CGR with missing sampling dates *gag* (n = 12) and *pol* (n = 12) treedater was provided with a broad range of sample time limits (1980–2015) and it returned a point estimate of the missing sampling dates (19). Trees were rooted using a subtype D outgroup (accessions K03454 and AY371157, for *gag* and *pol* respectively) (19).

### Phylodynamic analysis

We subdivided the large *gag* and *pol* phylogenies into partitions with significant differences in coalescent patterns using *treestructure* (6). The partitions of the phylogeny that this method detects have different evolutionary patterns that are usually a result of different transmission patterns or geographic structure. The minimum clade size for partitions was set to 1000 tips. We used the significance level of α = 0.05 for finding a new split within a set of tips (6) and conducted a sensitivity analysis around the α parameter. We carried out a matching analysis to examine the similarity of grouping of sequence IDs in partitions across the two genes. The partition numbering is arbitrary, but we would expect individuals to group together across genes, in the absence of recombination.

For each partition, we estimated effective population size through time (Ne(t)) using the mlesky model (Maximum likelihood phylodynamic inference) R package version 4.0.5 to determine whether Ne(t) differed between partitions (25). We removed all the CGR sequences and other previous Botswana HIV cohorts from the phylogenies before we estimated the effective population size as non-BCPP sequences did not have associated demographic data. Ne(t) was estimated using precision parameter τ = 10 which is optimised within the function to control the smoothness of the estimated trajectories as there was no a *priori* details for the volatility of the trajectories (6). We also tested the robustness of our findings with precision parameters τ = 1 and τ = 20 to control for the smoothness by accounting for the sensitivity of out of sample prediction error (24). To account for the uncertainty in tree dating, we repeated the entire phylodynamic analysis across 100 bootstrap replicate phylogenies for each gene, each time re-estimating Ne(t).

## Statistical analysis

We examined partitions for differences in their demographic (age, sex, sample date), clinical (treatment status, inferred stage of infection) and geographical (districts) characteristics using χ2 tests, ANOVA and Tukey analysis in R v4.0.5. The 30 villages were categorized according to their districts, resulting in seven districts: Central, Kgatleng, Kweneng, Ngamiland, North East, South East, and Southern (Table S1). We sought to identify characteristics associated with partitions with higher Ne(t) growth. We also determined the distribution of CGR sequences across the different partitions in the two genes.

## Results

### Both trees split into three partitions with divergent coalescent patterns

The time-resolved phylogenies were reconstructed for *gag* (5,493) and *pol* (4,965) HIV-1 subtype C sequences from Botswana, 2013 to 2018, inclusive of CGR sequences and other sequences from previous Botswana HIV cohorts. There is 97.5% similarity in sequence IDs between the *pol* and *gag* alignments after removal of CGRs and sequences from previous Botswana cohorts. To determine the time of common ancestor within Botswana for each gene, we removed the CGR sequences, then extracted the date of the root of each tree. Based on our analysis, the time of common ancestor in the Botswana *gag* phylogeny was 1979.9 (95% CI 1979.4–1980.4) and 1981.4 (95% CI 1980.0–1982.7) in *pol* phylogeny. We also determined the time of common ancestor including the CGR sequences, yielding 1970.3 (95% CI 1969.0–1971.6) for the *gag* phylogeny and 1956.8 (95% CI 1955.1 −1958.5) for *pol*. Previous estimates of time of common ancestor for HIV-1 subtype C range around 1960.4 (95% CI: 1950.9–1968.5) for *gag* and 1958.4 (95% CI: 1951.8–1964.9) for *pol* (30).

*Treestructure* produced three partitions in each of the HIV-1 subtype C gene phylogenies. Each tip represents a participant sample from a single time point and the average sample dates were identical across all partitions (Table 2). In the *gag* phylogeny all three partitions comprised more than 1,000 sequences inclusive of CGRs. In the *pol* phylogeny, two partitions were greater than 1,000 in size whereas the third partition comprised only 967 sequences inclusive of CGR sequences (Table 2).

We looked at the similarity in the grouping of the same sequence IDs across *gag* and *pol* partitions. Overlap is expected as sequence IDs are expected to group similarly across the two genes for the sequence IDs having both *gag* and *pol* gene sequences. The matching analysis showed an overlap in the distribution of sequence IDs across the partitions in *gag* and *pol* of 60.8% (Table 1).

### The partitions have distinct historical dynamics

**Figure 1:**
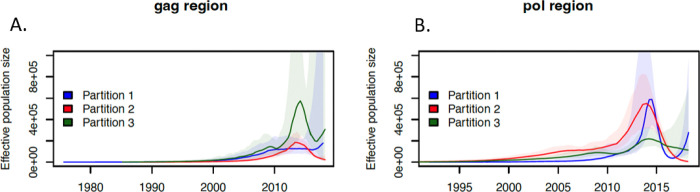
Estimated effective population size though time for three partitions in the Botswana *gag* and *pol* HIV-1 phylogenies. The N_e_(t) was estimated using the mlesky method with precision parameter t= 10 and the 95% CI for N_e_(t) are shaded.

The *mlesky* model revealed different patterns of temporal variation in effective population size Ne(t) across the three partitions in each phylogeny. In *gag*, partition 1 showed a slight increase in Ne(t) post-2000, remaining stable after 2010 (Fig. 1A). Partitions 2 and 3 showed an increase in Ne(t) after 2010, then a decline post-2015. The increase and decline were steeper for partition 3 than for partition 2. Partition 1 and 3 show a distinct increase post-2015. As a sensitivity analysis, we re-estimated Ne(t) dynamics using precision parameter τ = 1 and 20 (Figure S2). Dynamics were remarkably consistent across cutoffs, although there is an increase post – 2015 with precision τ = 1 which is not present with τ = 20. Smaller values for τ are more likely to yield incorrect population size fluctuations (25), and so we did not give this increase any more consideration.

In the *pol* phylogeny, the three partitions showed an increase in Ne(t) from 2000 onwards with partition 1 and 2 showing a steep increase after 2010 (Fig. 1B). Partitions 1 and 2 experienced a steep decrease in Ne(t) post – 2015, then Ne(t) in partition 1 increased again post-2015. Partition 3 had a slight incline until 2015 then a constant or declining effective population size.

### The partitions display distinct demographic and clinical characteristics.

Across the *gag* dataset, 9.3% (n = 388) individuals were classified as recent and 90.7% (n = 3770) as chronic. For *gag*, partition 1 had ongoing growth, and relatively less treatment coverage compared to partition 2 (Table 3). Partition 3 had more recent infections (p = 0.030, Table 3) compared to other partitions, less treatment coverage, and was characterised by a sharp increase in Ne(t) post-2010 which remains high despite a dramatic decrease post-2015. Partition 2 had more PLWH on treatment (p = 0.002) and more chronically infected participants (p = 0.033).

Across the *pol* dataset, 9.6% (n = 391) individuals were classified as recent and 90.4% (n = 3679) as chronic. In *pol*, partition 1 and 3 had the highest Ne(t) at present. Partition 3 comprised more PLWH who were recently infected (p = 0.020, Table 3) and less PLWH on treatment compared to the other two partitions, thus patterns across *gag* and *pol* were consistent. Partitions 1 and 2 showed a steep increase in Ne(t) post-2010 and dramatic decrease in Ne(t) post-2015 when compared to partition 3. Partition 1, which showed a decline after 2015, had more PLWH who were on treatment (p = 0.001) and who were chronically infected (p = 0.001). There was no significant difference in age, gender, or sample date among partitions in either phylogeny. When we compared the geographic makeup of partitions, we found a difference in the distribution of districts across partitions by ANOVA and Tukey analysis (Table 2, Table S2, Figure S1). In *gag*, participants from Southern and Kweneng were distributed non-randomly across partitions (p = 0.012, Figure S1 panel B, comparison of districts 6 and 7). Further analysis using Tukey showed *gag* partition 1 had more PLWH coming from the Southern district (p = 0.020, Table 2 & Table S2). In *pol*, three pairs of districts showed patterns distinct from each other: South East and Central (p = 0.030, districts 2 and 1), Southern and Central (p = 0.001, districts 7 and 1) and Southern and North East (p = 0.020, districts 7 and 4). The Tukey test was employed to determine which partitions were associated with which districts, revealing that PLWH in partition 1 were mostly coming from Southern district (p = 0.001) and in partition 3, from South East district (p = 0.008) in the *pol* phylogeny. In summary, partitions with increasing effective population size and low ART coverage were more likely to include participants from the Southern and South East districts of Botswana. We fitted a multivariate model to our data to determine if differences in districts account for the difference in recency according to the different partitions. In the *gag* phylogeny, geographical districts (p = 0.002) and recency (p = 0.005) were significantly associated with phylogenetic partitions while their combined interaction (p = 0.114) was not significant. Similarly, in the *pol* phylogeny, the model indicated a differential distribution of districts (p = 0.006) and recent infections (p = 0.009) across partitions but their interaction (p = 0.842) was not significant. In conclusion, in a multivariate model, the effect of geography and recency were independent and their interaction was not significant.

### Sensitivity analysis

We conducted a sensitivity analysis on the cutoff for identifying partitions within the *gag* and *pol* phylogenies, assessing the impact of changing the α parameter from 5–1% or 10%. The g*ag* phylogeny split into 3 partitions across all the significance levels, however, partitions 1 and 2 swap with α = 0.1 and α = 0.01. The *pol* phylogeny split into 3 partitions except when α = 0.01, where only two partitions were identified (Table S4 and Figure S3). Epidemiological findings were not identical across thresholds; however, partitions displaying growth were consistently more likely to comprise sequences from recent infections and PLWH who were not on treatment (Table S4 and S5).

## Discussion

Our phylodynamic analysis revealed hidden population structure among HIV sequences in Botswana. Although Botswana has made tremendous progress in treating and managing HIV infections (9), HIV prevalence and incidence remain high. A detailed understanding of underlying HIV transmission patterns can help inform where effective public health interventions should be directed. Our analysis showed a reduction in Ne(t) after 2015, indicating a decrease in incidence following the introduction of universal treatment in Botswana. Despite a successful treatment programme (1), our analysis highlighted disparities in access to treatment in partitions with ongoing growth. Both trees had faster growing partitions characterised by recently infected individuals and less treatment coverage. This might suggest that new infections are driven by poor linkage to ART care (26) despite the inclusive ART eligibility which was rolled out in 2016 and 2019 (1, 3, 9).

Two districts, Southern and South East, were consistently overrepresented in fast-growing partitions with recent infections and low ART coverage. A previous analysis examining the HIV population structure and dynamics in Tennessee in the US (6) found some effect of geography on genetic clustering with evidence of geographic bridging between regions (27). However, the generalised epidemic HIV transmission patterns in Botswana likely differ from the US setting, molecular clusters are smaller with highly diversified mixing of communities (28). The Southern and South East districts are peri-urban adjacent to the capital city of Botswana where there is high mobility due to work opportunities and higher education pursuits. The South East district includes the capital city of Botswana, but importantly, this study did not sample PLWH from the capital. Our results are in accordance with those of Magosi et al (2022), who, in this same cohort, estimated that the proportion of HIV transmissions was highest in the South East region (inclusive of Southern and South East districts) of Botswana when compared with other regions (29). While our study identified districts with increased HIV transmission and would support regionally targeted interventions, phylogeographic analyses of HIV in Botswana have highlighted high intercommunity mixing of HIV-1 viral lineages (28). As a result of the high mobility of people in Botswana across districts, possibly due to the connection to highways and high HIV prevalence of communities/villages near the highways, country-wide public health interventions may provide more benefit to Botswana.

Our study had some limitations. Partitioning was not fully consistent across gene regions. This anomaly could be attributed to varying sample availability and sizes across the two genes (19) recombination and phylogenetic error. Not all PLWH in the BCPP study were sequenced, reducing the viral sequences pool to be analysed, so some population structure patterns of the Botswana epidemic may remain obscured; however, *treestructure* has been shown to perform well even in smaller phylogenies (6).

In conclusion, this work highlights the possibility of uncovering distinct HIV transmission patterns and demographic processes from phylogenetic analysis and phylodynamic modelling (6). Phylodynamic analysis suggests transmissions may be slowing in segments of the population with high coverage of ART. The exceptional quality of the BCPP dataset provided a rare opportunity for a phylodynamic analysis from randomly sampled genetic data to investigate the national-level population structure of a sub-Saharan HIV epidemic. Our results highlight the value in developing a national HIV genetic surveillance program in Botswana for continuing to detect sub-epidemics and identifying those most vulnerable to HIV acquisition who are usually missed (28).

## Figures and Tables

**Table 1:** Sequence Overlap in partitions.

	Gag Partition 1	Gag Partition 2	Gag Partition 3
Pol Partition 1	1404	146	290
Pol Partition 2	432	832	556
Pol Partition 3	309	72	550

Notes: Overlap in sequence IDs across partitions was 60.8%. The partitions were renamed to match based on the overlap analysis. Partitions 1 overlap by 75.3%, partitions 2 by 41.1% and partitions 3 by 51.1%.

**Table 2:** Demographic characteristics across three partitions in the HIV-1 *gag* and *pol* phylogenies.

gene	Partition ID	CGR	Total sequences	Female (%)	Male (%)	Age (mean)	Sample date (mean)	District: South East (%)	District: Southern (%)	p-value (partitions and districts)
*gag*	1	241	1156	67.5	28.4	40.3	2016.2	0.8	12	**(1&2) .020**
*gag*	2	293	2265	70.4	27.3	39.9	2016.2	0.6	7.9	
*gag*	3	302	1573	67.7	28.5	39.6	2016.2	1	11.1	
*pol*	1	34	1760	70.8	28.3	40	2016.2	0.3	7.2	**(1&2) .001**
*pol*	2	244	1791	71.4	27.1	39.6	2016.2	0.8	12.3	
*pol*	3	170	967	67.6	31	40.4	2016.3	1.4	11.2	**(1&3) .008**

Notes. The differences in demographic characteristics across partitions were compared using the Pearson χ^2^, using raw numbers for sex and number of close global references (CGR). Age, mean sample dates and district composition were analysed using ANOVA. Significant values from Pearson χ^2^ were further analysed with Bonferroni post hoc to determine which groups in each partition diverged. No further analysis was done on non-significant p-values and these were not included in Table 2. For the analysis of districts, the ANOVA test was followed by a Tukey test to identify diverging pairs, with significant values shown in bold in Table 2. Percentages were calculated excluding CGR sequences. Other districts are shown in Table S2.

**Table 3:** Clinical characteristics across three partitions in the HIV-1 *gag* and *pol* phylogenies

		Stage of Infection (%)		p-value	Treatment Status (%)		p-value
Gene	Partition ID	Chronic	Recent		No	Yes	
*gag*	1	90.5	9.5		21.0	74.2	
*gag*	2	92.0	8.0	**.033**	17.1	80.1	**.002**
*gag*	3	88.7	11.3	**.030**	21.2	74.4	
*pol*	1	92.4	7.6	**.001**	16.2	82.4	**.001**
*pol*	2	88.9	11.1		21.2	76.3	
*pol*	3	88.8	11.2	**.020**	23.3	75	

Notes. Categorical variables (stage of infection and treatment status) were compared across partitions by the Pearson χ^2^ using raw numbers. The significant values were further analysed with Bonferroni post hoc to identify which groups within each partition were significant. p-values below 0.05 are shown in bold.

## Data Availability

The R and python scripts used in our analyses are available on GitHub (https://github.com/kkotokwe-bot/pangeabot). The BCPP data and protocols are publicly available (https://data.cdc.gov/Global-Health/Botswana-Combination-Prevention-Project-BCPP-Publi/qcw5-4m9q). HIV sequences and basic demographic and clinical data are available upon request to the PANGEA consortium (https://www.pangea-hiv.org/).

## Abbreviations

BCPP: Botswana Combination Prevention Project
ART: Antiretroviral Therapy
PLWH: People living with HIV
CGR: Close Global References
NGS: Next Generation Sequencing
Mlesky: Maximum likelihood phylodynamic inference
PEPFAR: President’s Emergency Plan for AIDS Relief
HRDC: Human Resource Development Council
HIV: Human Immunodeficiency Virus
